# Raloxifene Prevents Early Periprosthetic Bone Loss for Postmenopausal Women after Uncemented Total Hip Arthroplasty: A Randomized Placebo‐Controlled Clinical Trial

**DOI:** 10.1111/os.12696

**Published:** 2020-07-19

**Authors:** Long Gong, Yao‐yao Zhang, Na Yang, Huan‐juan Qian, Ling‐kun Zhang, Ming‐sheng Tan

**Affiliations:** ^1^ Department of Orthopaedic Surgery China–Japan Friendship Hospital, Peking Union Medical College, Chinese Academy of Medical College Beijing China; ^2^ Department of Obstetrics and Gynecology West China Second University Hospital of Sichuan University Chengdu China; ^3^ Key Laboratory of Birth Defects and Related Diseases of Women and Children of the Ministry of Education Chengdu China; ^4^ Bao Ding Maternal and Children Hospital Baoding China; ^5^ Department of Orthopedics Surgery 81 Group Military Hospital of Chinese PLA Baoding China

**Keywords:** Bone loss, Postmenopausal osteoporosis, Raloxifene, Total hip arthroplasty (THA)

## Abstract

**Objective:**

To examine the results of raloxifene for prevention of periprosthetic bone loss around the femoral stem in patients undergoing total hip arthroplasty (THA).

**Methods:**

Between January 2015 and May 2017, 240 female patients between 55 and 80 years underwent primary THA and were randomly allocated to receive 60 mg raloxifene hydrochloride per day (treatment group, TG, n = 120) or placebo (control group, CG, n = 120) orally at bedtime using computer‐generated randomization sequence generation. Baseline data, the Western Ontario McMaster Universities Osteoarthritis Index (WOMAC), women's quality of life (QoL) score, bone mineral density (BMD) around the prosthesis, and adverse events were compared between the two groups. The measuring range of BMD around the prosthesis was divided into seven regions of interest (ROI). The sample size was calculated to detect a mean difference in BMD of 0.15 g/cm^2^ with a standard deviation (SD) of 0.3. The error was set at 0.05 and the power level at 90% with additional compensation for a possible dropout rate of 20%.

**Results:**

A total of 240 participants in the study up to 24 months after THA. There were no significant differences in the mean BMD of all the zones between groups before surgery (all *P* > 0.05). However, there were significant differences in the BMD of Gruen zones 4 and 7 between groups at 6 months postoperatively (both *P* < 0.05); there were significant differences in Gruen zones 1, 4, 6, and 7 at 12 months postoperatively (all *P* < 0.01); there were significant differences in Gruen zones 1, 2, 4, 6, and 7 at 24 months postoperatively (all *P* < 0.001). Patients taking raloxifene reported higher QoL scores, with better improvement in BMD in all areas except in zones 3 and 5 compared with the control group. There were no significant differences in WOMAC pain (*P* = 0.4045), WOMAC function (*P* = 0.4456) and women's QoL scores (*P* = 0.5983) between groups before surgery. However, WOMAC pain, WOMAC function and women's QoL score in the treatment group were significantly better at all time points (all *P* < 0.05). Patients in the treatment group showed no increased adverse events, including cardiac events, stroke, venous thromboembolism, and gynecological cancer (all *P* > 0.05), but did show decreased odds of breast cancer in comparison with those using a placebo (*P* = 0.0437).

**Conclusion:**

Raloxifene can help inhibit bone loss around the prosthesis and improve the QoL of postmenopausal women after THA with no increased adverse events, and can even decrease the odds of breast cancer.

## Introduction

Total hip arthroplasty (THA) can restore hip function to significantly improve the quality of life (QoL)^1^, ^2^. More than 230,000 patients in the United States alone and approximately 500,000 patients worldwide undergo THA surgery every year^3^. These figures increase by approximately 10% per year^4^, ^5^. Unfortunately, the joint revision rate due to aseptic loosening of prostheses is as high as 12%^6^. As the average age of patients undergoing primary THA is 53.5 to 63 years old, a considerable portion of these patients are postmenopausal women who have a higher risk of osteoporosis^1^, ^2^.

Recent studies have indicated that female patients with low systemic bone mineral density (BMD) show greater bone loss in parts of the zone around the prosthesis after cementless THA than patients with normal BMD^6^, ^7^. Low BMD contributes to advanced implant migration in the early stage after THA, with worse initial stability and delayed osseointegration^8^, ^9^. Osseointegration is a prerequisite for successful implants^9^. Although it remains unclear whether or not patients with osteoporosis would suffer a higher risk of aseptic loosening, it is caused, or at least accompanied, by bone loss around femoral stem components.

Although many studies have tried reducing periprosthetic bone loss through drug therapy, at present, a standardized treatment regimen is not clear. Attention has been paid to bisphosphonates^10^, because this drug benefits the preservation of periprosthetic bone for postmenopausal osteoporosis in most cases^2^. However, there are numerous side effects, such as gastrointestinal disturbances^2^. These side effects limit its use in some particular populations.

Raloxifene, a nonsteroidal selective estrogen‐receptor modulator (SERM), has been proved to be an effective drug in the prevention and treatment of postmenopausal osteoporosis^10^, ^11^. In addition to contributing to reducing bone loss and increasing BMD, raloxifene has extra benefits with its unique advantages in postmenopausal women: (i) lowering the risk of invasive breast cancer in women at high risk of breast cancer; (ii) decreasing plasma concentration of lipoprotein, inflammatory factors, and oxidative stress; (iii) benefiting cognition, depression, sleep, and sexual function, despite being controversial^11^, ^12^, ^13^. Some of these problems, such as sleep disorder and hyperlipidemia, are extremely common in postmenopausal women. Therefore, raloxifene has become the first‐line drug for treating osteoporosis in postmenopausal women^11^, ^13^. However, to the best of our knowledge, no study has investigated whether raloxifene prevents periprosthetic bone loss around the implant. Therefore, we explore the use of drug therapy to prevent periprosthetic bone loss.

The purpose of this study is: (i) to evaluate the effects of raloxifene on periprosthetic bone loss in postmenopausal women after uncemented total hip arthroplasty; (ii) to assess whether raloxifene has additional benefits for postmenopausal women, such as improving their quality of life and reducing breast cancer risk; and (iii) to discuss the superiority in application of this anti‐osteoporosis drug.

## Methods

This was a prospective, randomized, double‐blind, controlled study conducted by a clinical team from January 2015 to May 2017. All staff and patients were blinded to the study. The study was approved by the institutional review board of the authors' affiliated institution (252–2015–023‐02), and informed consent was obtained from the participants.

### *Inclusion and Exclusion Critieria*

Inclusion criteria were women who: (i) were aged between 55 and 80 years with hip fractures, osteoarthritis, or femoral head necrosis; (ii) had undergone primary THA; and (iii) had completed records about the quality of Life score, BMD, adverse events, and complications.

We excluded those patients who: (i) had neurological diseases, mental disorders, and affective disorders; (ii) suffered from severe cardiac, renal, or hepatic dysfunctions; (iii) had severe metabolic, endocrine, electrolyte disturbances, or venous thromboembolic disease; (iv) had a history of unexplained vaginal bleeding or spotting, endometrial hyperplasia, gynecological tumors or breast cancer; (v) had received treatment including bisphosphonates, vitamin D, calcium, or calcitonin within 1 year from enrollment, as well as patients on sex hormone replacement therapy (HRT) or any other medication that may affect bone metabolism; and (vi) had an underlying bone disorder such as Paget's disease, osteogenesis imperfecta, rheumatoid arthritis, systemic lupus erythematosus, and other collagen vascular diseases. Using these criteria, 240 patients were included. Demographic data and baseline information are detailed in Table 1.

**Table 1:** Demographic characteristics and baseline information (mean ± SD)

Variables	Treatment group (*n* = 120)	Control group (*n* = 120)	*P*‐value
Patient characteristics			
Age (years)	62.5 ± 5.4	63.2 ± 5.0	0.2985
BMI (kg/m^2^)	26.8 ± 2.8	27.0 ± 3.0	0.5939
Preoperative data			
Intraoperative blood loss (mL)	115.2 ± 30.2	120.4 ± 32.5	0.2182
Duration of surgery (min)	56.8 ± 10.5	59.0 ± 11.5	0.1230

BMI, body mass index.

### *Intervention*

Participants were randomly allocated to receive 60 mg raloxifene hydrochloride per day (treatment group, TG, n = 120) or placebo (control group, CG, n = 120) orally at bedtime using a computer‐generated randomization sequence (SAS Statistical Software 9.1.3).

**Figure 1:**
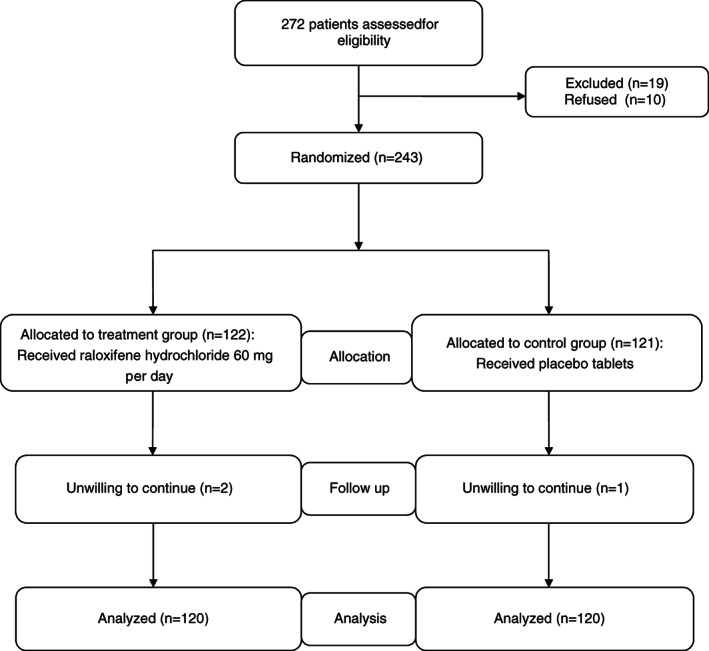
Flowchart of enrolled patients.

Participants were prescribed raloxifene hydrochloride (Eli Lilly, USA) or placebo either for at least 2 years postoperatively, with a high adherence (medication possession ratio > 90%, calculated as the number of daily doses received divided by the number of days of follow‐up), or if at least 22 prescriptions had been filled in the 2 years after treatment initiation. Participants were asked not to receive any other specific intervention for osteoporosis. All participants received supervision from two experienced endocrinologists at least once per month. Upon the absence of severe adverse events, the participant was asked not to continue the following steps. The flow of participants through each stage of the study is presented in Fig. 1.

### *Outcome Measures*

#### *Quality of Life*

The Western Ontario McMaster Universities Osteoarthritis Index (WOMAC) was used as a self‐reporting measure to evaluate QoL. The WOMAC Osteoarthritis Index, a reliable and valid disease‐specific questionnaire for assessing THA outcomes, was used to measure hip pain and function^14^. Two domains reflect joint function and pain. A lower score represents better recovery.

The women's QoL questionnaire in this study is designed to assess physical and emotional wellbeing in older women. We used a modified version to capture eight components of women's health, including depressed mood, somatic symptoms, memory and concentration, vasomotor symptoms, anxiety or fears, sexual function, sleep problems, and menstrual symptoms^15^. Each item was rated on a four‐point scale: “yes, definitely,” “yes, sometimes,” “no, not much,” and “no, not at all,” and this was reduced to a binary scale; that is, 1 and 2 (coded 0) *vs* 3 and 4 (coded 1) for scoring. A higher score represents better QoL.

#### *The Measuring Method of Bone Mineral Density*

The bond mineral density in this study was measured using bone density meter of lunar dual‐energy X‐ray (GE, USA) and orthopaedic analyzing software of Lunar Orthotm. The correcting test was performed before each measurement for a variation coefficient of less than 1%. Peak values of BMD were referenced from the data for female adults supported by the World Health Organization (WHO). During the measuring process, the lower limb should be rotated at 15°.

**Figure 2:**
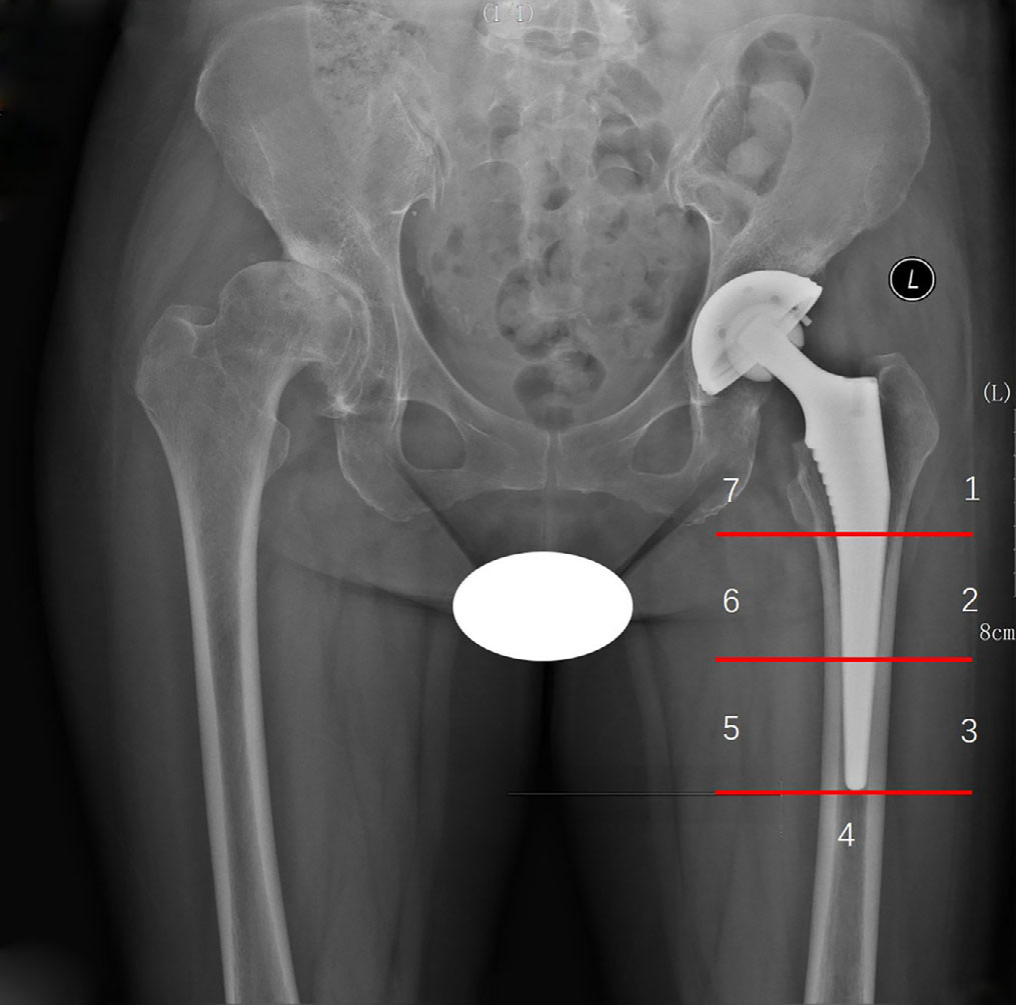
The defined Gruen zones of the periprosthetic side. The length from the upper edge of the prosthesis stem's medial side to its distal end is divided into three equal parts. The 2‐cm area of the distal end of the prosthesis is zone 4, and the outer and inner side of the prosthesis from top to bottom is, respectively, zone 1 to 3 and zone 5 to 7.

The measuring range of BMD around the prosthesis is divided into seven regions of interest (ROI) according to a previous study^16^. The precision error varied from 1.5% to 3.4% depending on the ROIs, with an average precision error of 2.3%^16^. The length from the upper edge of the prosthesis stem's medial side to its distal end is divided into three equal parts. The 2‐cm area of the distal end of the prosthesis is zone 4, and the outer and inner side of the prosthesis from top to bottom is, respectively, zones 1 to 3 and zones 5 to 7 (Fig. 2).

#### *Perioperative Management Protocol*

Total hip arthroplasty was performed using a standard Watson–Jones approach with a fully coated tapered stem (LCU Hip system, Waldemar Link, Munich, Germany) and acetabular cup (CombiCup, Waldemar Link, Munich, Germany). Both the acetabular cup and the femoral stem were cementless. All patients were routinely given prophylactic cefuroxime 1 hour preoperatively and in the first 24 hours postoperatively (1.5g, iv, tid). Preventive anticoagulant therapy (10 mg rivaroxaban every day or 2850 international units [IU] low‐molecular‐weight heparin [LMWH] [body weight < 90 kg] or 5700 IU [bodyweight >90 kg]) began within 12 hours postoperatively and continued for at least 28 days. Patients were encouraged to begin weight‐bearing as soon as tolerable with the help of ambulatory aids (usually within the first 24 hours) and were then allowed to discontinue the assistance of aids as they could ambulate without a limp (usually within 6–12 weeks). The physical therapist performed a functional exercise for all patients. After THA, patients were required to be subsequently followed up 6 and 12 months and 2 years postoperatively.

The postoperative X‐ray alignment on standard views was evaluated by three experienced orthopaedists and all enrolled cases had good quality component placement without the femoral stems placed in a varus or valgus position.

### *Sample Size*

The sample size was calculated to detect a mean difference in BMD of 0.15 g/cm^2^, with a standard deviation (SD) of 0.3. The error was set at 0.05 and the power level at 90%, with additional compensation for a possible dropout rate of 20%. The required sample size was 50 patients in each group, at least.

### *Statistical Analysis*

Countable variables were presented as percentages and compared using the χ^2^‐test. Normally distributed continuous and non‐normally distributed continuous data were respectively presented as mean ± SD and the median and range. A *t*‐test was used to compare clinical outcomes between groups. The analyses of the changes in BMD and bone markers were conducted based on the intention‐to‐treat (ITT) principle. *P* < 0.05 was considered statistically significant and power analysis was ≤ 0.9. All data were collected and analyzed using SAS 9.1 (SAS Institute, Cary, NC, USA).

## Results

### *General Results*

Based on inclusion and exclusion criteria, 240 patients were included in this study and completed the study protocol from January 2015 to May 2017. Baseline data are detailed in Table 1 and they were similar in the two groups (Tables 1, 2, 3).

**Table 2:** Quality of life scores between two groups at different time points

	Baseline			6 months postoperatively			12 months postoperatively			24 months postoperatively		
	TG (*N* = 120)	CG (*N* = 120)	*P*‐value	TG (*N* = 120)	CG (*N* = 120)	*P*‐value	TG (*N* = 120)	CG (*N* = 120)	*P*‐value	TG (*N* = 120)	CG (*N* = 120)	*P*‐value
*WOMAC*												
Mean pain score	37.2 (11.6)	38.5 (12.5)	0.4045	20.6 (10.0)	24.6 (9.8)	0.0020*	10.5 (6.7)	15.6 (7.4)	<0.0001*	7.5 (6.2)	12.6 (7.0)	<0.0001*
Mean function score	80.2 (18.5)	82.1 (20.0)	0.4456	60.5 (12.2)	68.2 (14.0)	<0.0001*	32.0 (10.0)	40.5 (12.2)	<0.0001*	24.0 (10.0)	30.5 (12.2)	<0.0001*
Women's *QoL*	16.8 (4.6)	16.5 (4.2)	0.5983	21.2 (4.5)	19.8 (4.4)	0.0156*	25.8 (4.0)	23.6 (4.2)	<0.0001*	29.0 (4.5)	26.3 (4.7)	<0.0001*

Values in parentheses are standard deviations. The student *t*‐test was used for statistical analysis.

* There was a statically significant difference between groups.

CG, control group; TG, treatment group; QoL, quality of life; WOMAC, Western Ontario McMaster Universities Osteoarthritis Index

**Table 3:** The differences in the bone mineral density (BMD) around the proximal femoral prostheses in each Gruen Zone at different time points

Gruen Zones	Baseline			6 months postoperatively			12 months postoperatively			24 months postoperatively		
	TG (*N* = 120)	CG (*N* = 120)	*P*‐value	TG (*N* = 120)	CG (*N* = 120)	*P*‐value	TG (*N* = 120)	CG (*N* = 120)	*P*‐value	TG (*N* = 120)	CG (*N* = 120)	*P*‐value
Zone 1	0.667 (0.23)	0.707 (0.15)	0.1119	1.100 (9.512)	−0.251 (9.232)	0.2653	7.235 (11.623)	2.321 (12.325)	0.0017*	12.520 (16.251)	3.121 (15.235)	<0.001*
Zone 2	1.383 (0.33)	1.413 (0.30)	0.4184	−2.655 (3.121)	−3.215 (4.265)	0.2469	−1.567 (5.625)	−2.985 (6.254)	0.0660	3.215 (7.256)	−2.865 (8.525)	<0.001*
Zone 3	1.434 (0.47)	1.450 (0.23)	0.7379	−2.625 (6.255)	−3.658 (5.258)	0.1695	−4.525 (7.852)	−6.258 (7.252)	0.0761	−3.202 (4.632)	−4.252 (5.052)	0.0940
Zone 4	1.567 (0.53)	1.621 (0.63)	0.4731	−2.362 (4.958)	−3.525 (3.658)	0.0394*	−0.765 (5.625)	−2.884 (5.471)	<0.001*	−0.655 (5.449)	−2.256 (4.872)	<0.001*
Zone 5	1.423 (0.45)	1.397 (0.51)	0.6758	−0.202 (5.250)	−0.700 (5.830)	0.4858	−0.987 (7.638)	−1.579 (6.882)	0.5288	−0.100 (6.852)	−1.620 (7.258)	0.0970
Zone 6	1.400 (0.38)	1.36 (0.42)	0.4399	−1.089 (4.855)	−1.985 (4.665)	0.1462	−0.765 (5.114)	−3.897 (4.825)	<0.001*	−0.555 (6.252)	−4.000 (7.005)	<0.001*
Zone 7	0.98 (0.25)	0.94 (0.36)	0.3185	7.656 (5.521)	−9.253 (6.825)	<0.001*	−8.878 (7.565)	−15.725 (6.877)	<0.001*	−9.885 (8.000)	−23.665 (7.120)	<0.001*

Values in parentheses are standard deviations. The student *t*‐test was used for statistical analysis. CG, control group; TG, treatment group.

* There was statically significant difference between groups.

### *Women's Quality of Life*

#### *Western Ontario McMaster Universities Osteoarthritis Index Pain Score*

There was no significant difference in the WOMAC pain score (37.2 ± 11.6 *vs* 38.5 ± 12.5, *P* = 0.4045) between groups before surgery. The mean WOMAC pain score at 6 months, 12 months, and 24 months postoperatively was 20.6 ± 10.0, 10.5 ± 6.7, and 7.5 ± 6.2 in the treatment group and 24.6 ± 9.8, 15.6 ± 7.4, and 12.6 ± 7.0 in the control group. The differences between groups at all time points were statistically significant (all *P* < 0.05) (Table 2). This score in the treatment group decreased by 19.4% (*P* = 0.002), 48.6% (*P* < 0.0001), and 68.0% (*P* < 0.0001) at 6 months, 12 months, and 24 months postoperatively, respectively, in comparison with that in the control group.

#### *Western Ontario McMaster Universities Osteoarthritis Index Function Score*

There was no significant difference in the WOMAC function score (80.2 ± 18.5 *vs* 82.1 ± 20.0, *P* = 0.4456) between groups before surgery. The mean WOMAC function score at 6 months, 12 months, and 24 months postoperatively was 60.5 ± 12.2, 32.0 ± 10.0, and 24.0 ± 10.0 in the treatment group and 68.2 ± 14.0, 40.5 ± 12.2, and 30.5 ± 12.2 in the control group. The differences between groups at all time points were statistically significant (all *P* < 0.0001) (Table 2). This score in the treatment group decreased by 12.7% (*P* < 0.0001), 26.6% (*P* < 0.0001), and 27.1% (*P* < 0.0001) at 6 months, 12 months, and 24 months postoperatively, respectively, in comparison with that in the control group.

#### *Women's Quality of Life Score*

There was no significant difference in women's QoL score (16.8 ± 4.6 *vs* 16.5 ± 4.2, *P* = 0.5983) between groups before surgery. The mean women's QoL score at 6 months, 12 months, and 24 months postoperatively was 21.2 ± 4.5, 25.8 ± 4.0, and 29.0 ± 4.5 in the treatment group and 19.8 ± 4.4, 23.6 ± 4.2, and 26.3 ± 4.7 in the control group. The differences between groups at all time points were statistically significant (all *P* < 0.05) (Table 2). This score in the treatment group decreased by 7.1% (*P* = 0.0156), 9.3% (*P* < 0.0001), and 10.3% (*P* < 0.0001) at 6 months, 12 months, and 24 months postoperatively, respectively, in comparison with that in the control group.

### *Periprosthetic Bone Mineral Density Changes*

#### *Gruen Zone 1*

The mean BMD before surgery, at 6 months, 12 months, and 24 months postoperatively was 0.667 ± 0.23, 1.100 ± 9.512, 7.235 ± 11.623, and 12.520 ± 16.251 in the treatment group and 0.707 ± 0.15, −0.251 ± 9.232, 2.321 ± 12.325, and 3.121 ± 15.235 in the control group (Table 3).

**Figure 3:**
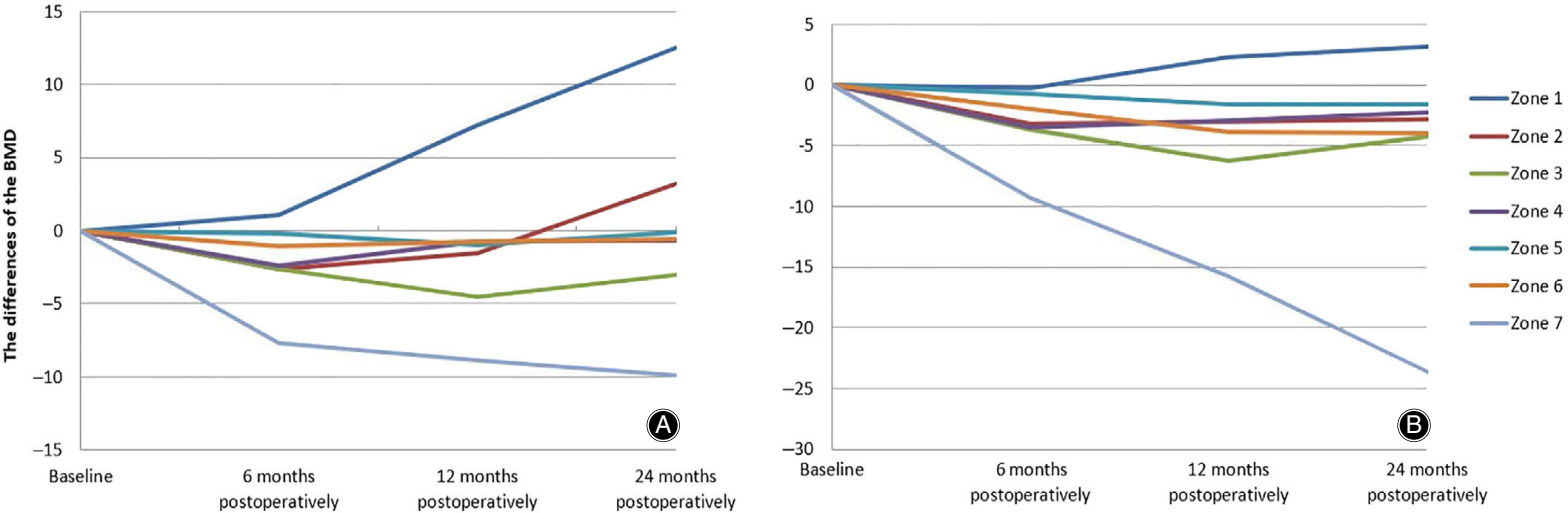
The differences in the change in bone mineral density (BMD) in each Gruen zone at different time points in the treatment group (A) and the control group (B). For patients in the treatment group, Gruen zones 1, 2, 4, and 6 showed a continuous increase in BMD; Gruen zones 3 and 5 showed a slight decrease but recovered thereafter; Gruen zone 7 showed a continuous decrease within 2 years postoperatively. In contrast, for patients in the control group, Gruen zones 1, 2, and 4 showed a slight increase; Gruen zone 5 showed a slight decrease; Gruen zone 3 showed a decline 12 months postoperatively but recovered thereafter; Gruen zones 6 and 7 showed a continuous decrease in the 2 years postoperatively.

Gruen zone 1 in the treatment group showed a continuous increase in the mean value of BMD from 1.100 to 12.521 during the 2 years postoperatively, while this zone showed a slight increase in BMD from −0.251 to 3.121 in the control group (Table 3, Fig. 3).

The mean BMD in zone 1 in the treatment group significantly increased by 211.7% (*P* = 0.0017) and 301.2% (*P* < 0.001), 12 months, and 24 months postoperatively, respectively, in comparison with those in the control group, while before surgery and 6 months postoperatively, there were no significant differences (both *P* > 0.05) (Table 3).

#### *Gruen Zone 2*

The mean BMD before surgery, at 6 months, 12 months, and 24 months postoperatively was 1.383 ± 0.33, −2.655 ± 3.121, −1.567 ± 5.625, and 3.215 ± 7.256 in the treatment group and 1.413 ± 0.30, −3.215 ± 4.265, −2.985 ± 6.254, and − 2.865 ± 8.525 in the control group (Table 3).

Gruen zone 2 in the treatment group showed a continuous increase in the mean value of BMD from −2.655 to 3.215 during the 2 years postoperatively, while this zone showed a slight increase in BMD from −3.215 to −2.865 in the control group (Table 3, Fig. 3).

The mean BMD in zone 2 in the treatment group significantly increased, by 212.2% (*P* < 0.001), 24 months postoperatively in comparison with that in the control group. For the rest of the time points, there were no significant differences (all *P*>0.05) (Table 3).

#### *Gruen Zone 3*

The mean BMD before surgery, at 6 months, 12 months, and 24 months postoperatively was 1.434 ± 0.47, −2.625 ± 6.255, −4.525 ± 7.852, and − 3.202 ± 4.632 in the treatment group and 1.450 ± 0.23, −3.658 ± 5.258, −6.258 ± 7.252, and − 4.252 ± 5.052 in the control group (Table 3).

Gruen zone 3 in the treatment group showed a slight decrease in the mean value of BMD from −2.625 to −4.525 during the 2 years postoperatively, while this zone showed a decline in BMD from −3.658 to −6.258 in the control group (Table 3, Fig. 3).

There were no significant differences in BMD in zone 3 between groups at all time points (all *P* > 0.05) (Table 3).

#### *Gruen Zone 4*

The mean BMD before surgery, at 6 months, 12 months, and 24 months postoperatively was 1.567 ± 0.53, −2.362 ± 4.958, −0.765 ± 5.625, and − 0.655 ± 5.449 in the treatment group and 1.621 ± 0.63, −3.525 ± 3.658, −2.884 ± 5.471, and − 2.256 ± 4.872 in the control group (Table 3).

For patients in the treatment group, Gruen zone 4 showed a continuous increase in the mean value of BMD from −2.362 to −0.655 during the 2 years postoperatively, while this zone showed a slight increase in BMD from −3.525 to −2.256 in the control group (Table 3, Fig. 3).

The mean BMD in zone 4 in the treatment group significantly increased, by 33.0% (*P* = 0.0394), 73.5% (*P* < 0.001), and 71.0% (*P* < 0.001), 6 months, 12 months, and 24 months postoperatively, respectively, in comparison with those in the control group (Table 3).

#### *Gruen Zone 5*

The mean BMD before surgery, at 6 months, 12 months, and 24 months postoperatively was 1.423 ± 0.45, −0.202 ± 5.250, −0.987 ± 7.638, and − 0.100 ± 6.852 in the treatment group and 1.397 ± 0.51, −0.700 ± 5.830, −1.579 ± 6.882, and − 1.620 ± 7.258 in the control group (Table 3).

For patients in the treatment group, Gruen zone 5 showed a slight decrease in the mean value of BMD, from −0.202 to −0.987, during the 2 years postoperatively, a similar situation occurred in the control group, with a decrease in the mean value of BMD from −0.700 to −1.620 (Table 3, Fig. 3).

There were no significant differences in BMD in zone 5 between groups at all time points (all *P* > 0.05) (Table 3).

#### *Gruen Zone 6*

The mean BMD before surgery, at 6 months, 12 months and 24 months postoperatively, was 1.400 ± 0.38, −1.089 ± 4.855, −0.765 ± 5.114, and − 0.555 ± 6.252 in the treatment group and 1.36 ± 0.42, −1.985 ± 4.665, −3.897 ± 4.825, and − 4.000 ± 7.005 in the control group (Table 3).

For patients in the treatment group, Gruen zone 6 showed a continuous increase in the mean value of BMD, from −2.362 to −0.555 during the 2 years postoperatively, while this zone showed a continuous decrease in BMD, from −4.000 to −23.665 in the control group (Table 3, Fig. 3).

The mean BMD in zone 6 in the treatment group significantly increased, by 80.4% (*P* < 0.001) and 86.1% (*P* < 0.001), 12 months and 24 months postoperatively, respectively, in comparison with those in the control group, while before surgery and 6 months postoperatively, there were no significant differences (both *P* > 0.05) (Table 3).

#### *Gruen Zone 7*

The mean BMD before surgery, at 6 months, 12 months, and 24 months postoperatively was 0.98 ± 0.25, 7.656 ± 5.521, −8.878 ± 7.565, and − 9.885 ± 8.000 in the treatment group and 0.94 ± 0.36, −9.253 ± 6.825, −15.725 ± 6.877, and − 23.665 ± 7.120 in the control group (Table 3).

For patients in the treatment group, Gruen zone 7 showed a continuous decrease to −9.885, from 7.656 in the following 2 years postoperatively; a smilar situation occurred in the control group, from −9.253 to −23.665 (Table 3, Fig. 3).

The mean BMD in zone 7 in the treatment group significantly increased, by 182.7% (*P* < 0.001), 43.5% (*P* < 0.001), and 58.2% (*P* < 0.001), 6 months, 12 months, and 24 months postoperatively, respectively, in comparison with those in the control group (Table 3).

### *Adverse Events*

No patient showed periprosthetic infection and radiographic signs of component loosening or periprosthetic osteolysis within the first year postoperatively. There were no significant differences in cardiac events (5 *vs* 4, *P* = 0.7340), stroke (2 *vs* 1, *P* = 0.5612), venous thromboembolism (0 *vs* 1, *P* = 0.3163), and gynecological cancer (1 *vs* 0, *P* = 0.3163) between groups, except breast cancer (4 *vs* 0, *P* = 0.0437).

Adverse events associated with gastrointestinal disturbances, such as arthralgia, flu‐like illness with symptoms of fatigue, fever, chills, malaise, and myalgia, were not present in both groups during the study period (*P* < 0.001).

## Discussion

Although studies have tried reducing periprosthetic bone loss using drug therapy, at present, a standardized treatment regimen is still absent. Llewellyn G reports that the most commonly used treatments for osteoporosis in the primary care setting are bisphosphonates and selective estrogen receptor modulators (SERM)^12^. Considerable attention has been paid to bisphosphonates. This drug benefits the preservation of periprosthetic bone for postmenopausal osteoporosis^17^, ^18^. Although bisphosphonates are safe drugs in most cases, they have numerous side effects, such as gastrointestinal disturbances, arthralgia, elevated erythrocyte sedimentation rate and C‐reactive protein, and flu‐like illness with symptoms of fatigue, fever, chills, malaise, and myalgia^17^. These side effects limit its use in some particular populations. Previous studies demonstrated that raloxifene had statistically significantly lower^18^ or similar rates^12^, ^19^ of fractures due to decreased BMD in comparison with bisphosphonates. Ellewellyn G. indicated that patients in the raloxifene group believed that their QoL had improved more and had higher treatment satisfaction than patients in the bisphosphonate group^12^. Consistent with previous findings, our results showed that patients taking raloxifene reported higher QoL scores and have greater improvement in BMD in all areas except zones 3 and 5 compared with the control group.

Our results showed that raloxifene showed a better effect in inhibiting bone loss in the proximal zones than bone loss in the distal zones. There were significant differences in Gruen zones 4 and 7 at 6 months postoperatively. The BMD's changes in Gruen zones 4 and 7 in the treatment group were 1.16% and 16.9% higher than in the control group. There were significant differences in Gruen zone 1, zone 4, zone 6, and zone 7 at 12 months postoperatively. The BMD's changes for Gruen zones 1, 4, 6, and 7 in the treatment group were 4.91%, 2.12%, 3.13%, and 6.85% higher than in the control group. There were significant differences in Gruen zone 1, zone 2, zone 4, zone 6, and zone 7 at 24 months postoperatively. The BMD's changes for Gruen zones 1, 2, 4, 6, and 7 in the treatment group were 9.40%, 6.08%, 1.601%, 3.445%, and 13.78% significantly higher than for the control group, while zones 3 and 5 were not significant.

Consistent with the results of Jessica J's study^6^, Gruen zone 7 had the most variation among seven zones. BMD in Gruen zone 7 in the treatment and control group decreased by 9.89% and 23.67%, respectively. Although the BMD in both groups fell to a different extent, the reduction in the treatment group was 2.4 times less than that in the control group. This may be closely related to stress shielding in this area^16^. Under natural circumstances, the pressure of the proximal femur is conducted downward through the calcar femorale^16^, ^20^. By contrast, the load after THA is transferred through the femoral stem^20^. As a result, the bone in Gruen zone 7 bears less load and thereafter bone mass decreased significantly according to Wolff's law of mechanics^20^. Mechanical stress inhibited osteoclast proliferation through decreasing RANKL/OPG gene expression^21^. Raloxifene can bind to estrogen receptors and inhibit the activity of osteoclasts and, thus, make the bone density increase^10^, ^12^. This could help explain why raloxifene has a positive effect on less reduction in zone 7 in the treatment group.

Despite no significant differences in zone 3 and zone 5 at 24 months postoperatively, we can investigate the differences between groups in more periprosthetic areas that reached a statistically significant level with the longer follow‐up. In bone, estrogen deficiency has been associated with accelerated osteoblast apoptosis and susceptibility to osteoporosis. Raloxifene can stimulate the activity of osteoblasts and exert direct protective effects on human osteoblasts^22^, ^23^. Therefore, we assume that raloxifene can help bone density increase theoretically and it is a matter of time. Considering effects both on osteoclasts and osteoblasts, a complex balance between them under some factors such as age, gender, and mechanical stress and bone remodeling around the implants^7^, ^24^, there could exist delayed effects of raloxifene. These may help explain why no significant differences in zone 3 and zone 5 were detected in the raloxifene group. However, specific mechanisms and longer observation periods are needed in biochemistry and pharmacology experiments.

Several studies have evaluated the effects of other anti‐osteoporosis drugs on the prevention of periprosthetic bone loss around the femoral stem, and some have found positive results^25^, ^26^, ^27^, ^28^. Muren *et al*.^25^ reported that the effect of risedronate on preventing periprosthetic bone loss postoperatively failed to be seen at 4 years with a cohort of 61 patients. Venesmaa *et al*.^26^ demonstrated that alendronate led to a significant reduction in periprosthetic bone loss after primary uncemented THA compared with the changes found in patients without therapy. However, in this study the follow‐up time (6 months) was short and the study population of 13 patients was too small to make any long‐term conclusions as to the prognosis for THA patients treated with alendronate. Tapaninen *et al*.^27^ conducted a study on alendronate with a longer follow‐up of 5 years, and revealed that it had a positive effect on BMD changes during the first 6 months postoperatively. However, subsequently, the 5‐year BMD changes were not significantly different between groups. Huang *et al*. (2017)^28^ showed that zoledronic acid (ZA), a third‐generation bisphosphonate, could effectively revert the loss of periprosthetic BMD, especially in the proximal femur (zones 1 and 7), which is similar to the present results. All these studies mentioned above did not evaluate patients' QoL after THA. However, QoL is the crucial component in evaluating the clinical oucomes after arthroplasty. In the present study, scores related to women's QoL at all time points in the treatment group were significantly better than their postoperative counterparts in the control group. Raloxifene can improve postmenopausal women's QoL by relieving symptoms, including aching joints and muscles, sleep disturbance, and mental problems^12^, ^13^, ^18^. These positive effects, such as the unique advantages of SERM, cannot be replaced by bisphosphonates.

Vitor reported that osteoporosis was related to a patient's age, postmenopausal status, and not having estrogen replacement therapy (ERT)^29^. Prieto‐Alhambra reported that ERT is associated with an almost 40% reduction in revision rates after a TKA/THA by matching 2700 HRT users to 8100 non‐users, observing for a median of 3.3 (1.5–6.1) years postoperatively^30^. This is an encouraging result for surgeons. However, the adverse effects were accompanied by its benefits, such as breast cancer and abnormal uterine bleeding. Fortunately, raloxifene, as a kind of SERM, has the potential to retain most of the beneficial effects of estrogen while avoiding most of the adverse effects^21^. Our results showed that using raloxifene for 2 years after THA did not increase adverse events, including cardiac events, stroke, venous thromboembolism, and gynecological cancer, and even decreased the odds of breast cancer, which is consistent with the descriptions in previous studies^18^, ^19^.

There were several limitations to this study. First, our study was designed to investigate periprosthetic BMD changes, which is a surrogate variable for prosthesis stability, subsequent implant loosening, and periprosthetic fractures^31^. The relevance and predictive value of this parameter remain uncertain. Therefore, a much longer follow‐up period is needed to confirm a relationship between them. Of note, our results are significant and consistent with previous reports. We observed that BMD in most areas around the prosthesis decreased statistically significantly during the 2 years postoperatively. It was suggested that the initial periprosthetic bone remodeling process was mainly completed in the first 12 postoperative months^31^, ^32^. Previous studies indicated that 1–2 years is an adequate follow‐up period for the evaluation of early‐stage periprosthetic bone remodeling^32^. Second, based on our clinical experience, estrogen should not be initiated within the first week after THA, because its adverse reactions may mask or aggravate the patient's condition,. In this study, estrogen was given at 2–3 weeks after THA depending on the patient's recovery. No related short‐term adverse reaction after THA was found in any patient, which may prove that the time of first use after THA was appropriate. However, this needs to be studied rigorously. Third, the optimum duration of the use of raloxifene and rebound effects from its withdrawal were not considered in the present study. Considering that it is a kind of receptor drug, it should produce a rebound effect. Therefore, what it includes and how to avoid it need to be studied further. Finally, this study only recruited a particular patient cohort, postmenopausal women, which limits its clinical application. These limitations open the door for future studies.

### *Conclusion*

Our results revealed that raloxifene hydrochloride, as a kind of selective estrogen receptor modulator (SERM), could help inhibit bone loss around the prosthesis and improve women's QoL after THA with no increased adverse events, as well as decrease the odds of breast cancer.

## Disclosure

The authors declare no conflict of interest.
